# Supplementary material for: Prevalence of Antimicrobial Resistance in Escherichia coli and Salmonella Species Isolates from Chickens in Live Bird Markets and Boot Swabs from Layer Farms in Timor-Leste
Source: Antibiotics (Basel). 2024 Jan 25;13(2):120. doi: 10.3390/antibiotics13020120 (PMC10885974; doi:10.3390/antibiotics13020120)
Supplement: Supplementary file 1 [file antibiotics-13-00120-s001.zip › antibiotics-2833252-supplementary.pdf]

Table S1: Number of live bird markets (LBM), number of cloacal swabs collected and type of chicken where cloacal swab samples were collected from in 13 municipalities.

Municipality	Number of LBM	Number of cloacal swabs	Type of chicken			
			Local chicken	Fighting cock	Broiler	Layer
Aileu	1	5	3	2	0	0
Ainaro	2	16	10	6	0	0
Baucau	3	57	45	12	0	0
Bobonaro	2	64	42	11	10	1
Covalima	3	33	24	9	0	0
Dili	4	68	56	4	7	1
Ermera	1	13	7	6	0	0
Lautem	1	6	4	2	0	0
Liquica	1	12	12	0	0	0
Manatuto	1	16	9	7	0	0
Manufahi	2	23	16	7	0	0
Oecusse	2	23	19	4	0	0
Viqueque	2	9	7	2	0	0
Total	25	345	254	72	17	2

Table S2: Prevalence of AMR for *E. coli* isolates from disk diffusion results, by the origin it was collected from. Layer chickens from live bird markets are excluded from this table because there were only 2 isolates.

Antimicrobial	Local chickens (%) (n=217)	Fighting cock (%) (n=60)	Layer Farms (%) (n=74)	Broiler (%) (n=16)
Ampicillin	13.4	21.7	23.0	43.8
Streptomycin	6.0	8.3	16.2	31.3
Tetracycline	12.9	18.3	48.6	50.0
Enrofloxacin	0.9	0	9.5	0
Trimethoprim/Sulphamethoxazole	6.5	8.3	24.3	37.5
Sulfisoxazole	8.8	16.7	29.7	62.5
Multi-drug resistance	4.6	10.0	20.3	43.8

Table S3: Phenotypic resistance profile by antimicrobial classes for multi-drug resistant *E. coli* (n=29) and *Salmonella* spp. (n=1) based on broth-based microdilution.

Number of antimicrobial classes	Phenotypic resistance profile by antimicrobial class	Number of *E. coli* isolates	Number of *Salmonella* spp. isolates
3	Penicillin, β-lactam/β-lactam inhibitor combination, sulfonamide	5	0
	Penicillin, β-lactam/β-lactam inhibitor combination, aminoglycoside	5	0
	Penicillin, β-lactam/β-lactam inhibitor combination, cephalosporin	3	0
	Penicillin, cephalosporin, sulfonamide	1	0
	Carbapenem, polymyxin, aminoglycoside	1	0
4	Penicillin, cephalosporin, monobactam, polymyxin	4	0
	Penicillin, β-lactam/β-lactam inhibitor combination, fluoroquinolone, sulfonamide	1	0
	Penicillin, β-lactam/β-lactam inhibitor combination, cephalosporin, phosphonic acid	1	0
5	Penicillin, β-lactam/β-lactam inhibitor combination, cephalosporin, monobactam, polymyxin	5	0
	Penicillin, cephalosporin, polymyxin, aminoglycoside, fluroquinolone	1	0
	Penicillin, carbapenem, polymyxin, aminoglycoside, sulfonamide	0	1
6	Penicillin, β-lactam/β-lactam inhibitor combination, cephalosporin, monobactam, polymyxin, sulfonamide	1	0
7	Penicillin, β-lactam/β-lactam inhibitor combination, cephalosporin, carbapenem, polymyxin, aminoglycoside, phosphonic acid	1	0

Table S4: Crude associations between origin of *E. coli* isolates and antimicrobial resistances from broth-based microdilution using mixed effect logistic regression models. Of the 205 *E. coli* isolates included in the analysis, 125 were from local chickens, 37 from fighting cocks, and 43 from layer farms. Broilers were not included because there were only 6 isolates.

Antimicrobial/Origin of isolate	Odds ratio (95% CI)	p value
Ampicillin-clavulanic acid		*0.812*
Local chicken	Ref	
Fighting cock	1.3 (0.5 - 3.4)	0.540
Layer Farm	0.9 (0.2 - 3.9)	0.923
Ampicillin		*0.489*
Local chicken	Ref	
Fighting cock	1.0 (0.4 - 2.7)	0.936
Layer Farm	2.2 (0.6 - 8.1)	0.234
Aztreonam		*0.612*
Local chicken	Ref	
Fighting cock	1.7 (0.4 - 7.8)	0.483
Layer Farm	0.5 (0.0 - 6.0)	0.581
Cefepime		*0.416*
Local chicken	Ref	
Fighting cock	2.3 (0.5 - 9.5)	0.267
Layer Farm	0.5 (0.0 - 7.4)	0.617
Cefixime		*0.146*
Local chicken	Ref	
Fighting cock	2.7 (0.8 - 9.5)	0.116
Layer Farm	0.4 (0.0 - 3.7)	0.430
Ceftazidime		*0.406*
Local chicken	Ref	
Fighting cock	2.6 (0.5 - 12.7)	0.234
Layer Farm	0.7 (0.1 - 7.6)	0.786
Ceftriaxone		*0.416*
Local chicken	Ref	
Fighting cock	2.3 (0.5 - 9.5)	0.267
Layer Farm	0.5 (0.0 - 7.4)	0.617
Cefuroxime		*0.317*
Local chicken	Ref	
Fighting cock	2.0 (0.5 - 7.7)	0.321
Layer Farm	3.1 (0.6 - 15.7)	0.169
Cephalexin		*0.082*
Local chicken	Ref	
Fighting cock	2.3 (0.4 - 14.5)	0.366
Layer Farm	5.4 (1.2 - 23.4)	**0.026**
Ciprofloxacin		***0.025***
Local chicken	Ref	
Fighting cock	N/A	N/A
Layer Farm	12.7 (1.4 - 117.2)	**0.025**

Colistin		*0.223*
Local chicken	Ref	
Fighting cock	3.3 (0.9 - 12.9)	0.083
Layer Farm	1.7 (0.2 - 13.8)	0.627
Gentamicin		*0.908*
Local chicken	Ref	
Fighting cock	1.4 (0.3 - 6.7)	0.666
Layer Farm	1.2 (0.1 - 15.2)	0.884
Levofloxacin		*0.146*
Local chicken	Ref	
Fighting cock	N/A	N/A
Layer Farm	6.0 (0.5 - 68.4)	0.146
Mecillinam		*0.340*
Local chicken	Ref	
Fighting cock	1.0 (0.3 - 3.4)	0.982
Layer Farm	0.2 (0.0 - 1.7)	0.146
Piperacillin		*0.515*
Local chicken	Ref	
Fighting cock	1.2 (0.4 - 3.5)	0.703
Layer Farm	2.3 (0.5 - 10.0)	0.255
Temocillin		*0.882*
Local chicken	Ref	
Fighting cock	1.3 (0.4 - 4.4)	0.626
Layer Farm	1.0 (0.2 - 5.5)	0.986
Tobramycin		*0.605*
Local chicken	Ref	
Fighting cock	2.1 (0.5 - 9.3)	0.321
Layer Farm	1.2 (0.2 - 6.3)	0.854
Trimethoprim-Sulfamethoxazole		***0.005***
Local chicken	Ref	
Fighting cock	1.4 (0.3 - 7.4)	0.713
Layer Farm	6.4 (2.0 - 20.2)	**0.002**

Overall likelihood ratio p-values are italicised and individual Wald p-values are non-italicised. P-values <0.05 are in bold.

Amikacin, ertapenem and meropenem were excluded from the analysis because all isolates were sensitive. Fosfomycin, imipenem, nitrofurantoin and piperacillin-tazobactam were excluded from the analysis due to insufficient resistant isolates.

N/A – These categories are not included, as there were no isolates originating from fighting cocks that were resistant to ciprofloxacin and levofloxacin
